# Improvement of Natural Polymeric Films Properties by Blend Formulation for Sustainable Active Food Packaging

**DOI:** 10.3390/polym15092231

**Published:** 2023-05-08

**Authors:** Emanuela Drago, Roberta Campardelli, Alberto Lagazzo, Giuseppe Firpo, Patrizia Perego

**Affiliations:** 1 Department of Civil, Chemical and Environmental Engineering (DICCA), Polytechnic School, University of Genoa, Via Opera Pia 15, 16145 Genoa, Italy; emanuela.drago@edu.unige.it (E.D.); alberto.lagazzo@unige.it (A.L.); p.perego@unige.it (P.P.); 2 Department of Physics, Nanomed Lab, University of Genoa, Via Dodecaneso 33, 16146 Genoa, Italy; giuseppe.firpo@unige.it

**Keywords:** biopolymers, zein, chitosan, green technologies, solvent casting, active packaging, biodegradable packaging, materials optimization, zero waste, circular economy

## Abstract

Active packaging manufactured with biopolymers extracted from agri-food waste is one of the most innovative and eco-sustainable strategies for maintaining food quality. However, biopolymers often present poor performances, which hinders their competitiveness compared with plastics. This work focused on developing and optimizing a natural polymeric blend produced by solvent casting based on zein and chitosan to improve the pure biopolymers’ properties. The best results were obtained by blending zein and chitosan in a 1:2 weight ratio. The films were characterized in terms of morphology, mechanical and oxygen barrier properties, thermal stability, transparency and wettability. The blend production allowed us to obtain lower brittleness and lower stiffness materials compared with pure polymer films, with oxygen permeability values two orders of magnitude lower than pure zein, better optical properties with respect to pure chitosan and good thermal stability. The wettability properties of the blend did not result in being altered with respect to the single polymer, which was found to have hydrophilic behavior, highlighting the strong influence of glycerol used as a plasticizer. The results suggested that the polymer blending strategy is a viable and cost-effective method for producing packaging materials as alternatives to plastics.

## 1. Introduction

In the food sector, plastic pollution is constantly increasing as a result of the high globalization of this century. In fact, the consumer requires more and more ready-to-use food products, often in single portions, with ever-increasing quality and freshness. This involves packaging materials with a more complex structure and, therefore, less recyclable as well as requiring lower cost of raw material. All these needs are met by the use of polymers of fossil origin [1]. To date, plastic is the most used material in the food packaging field because it satisfies many properties necessary for good packaging, such as lightness, good mechanical properties, barrier properties to gases and vapors and excellent transparency and flexibility, with costs lower than other materials. However, not all types of plastic guarantee chemical inertness with food, and moreover, plastic objects have an evident environmental impact once their cycle of use has ended. Therefore, in recent years new methods have been sought to create packaging that has the same characteristics as plastic material but is composed of green, ecological and eco-sustainable material in accordance with the zero-waste objective set by the 2030 Agenda [2].

Among the natural polymers, zein is a protein obtained from corn processing waste, whose recovery is of interest to the circular economy. It is a protein with a strong hydrophobic character and consequent insolubility in water due to a large amount of non-polar amino acids present in its structure. Furthermore, it is low in essential amino acids and does not have a high nutritional quality for human consumption [3]. The applications of this protein are not limited to the packaging field but also extend to the biomedical, pharmaceutical and textile ones. Some examples of these applications are the production of zein nanoparticles loaded with drugs for their controlled release [4] or the manufacture of zein-based antimicrobial fibers as an alternative to synthetic textile materials [5].

However, focusing on the food packaging field, the high hydrophobicity of zein can lead to some issues together with other characteristics that hinder zein-based film production on a large scale, such as its characteristic yellow color [3]. Another disadvantage of zein is represented by the poor mechanical properties that these materials can offer, far from those offered by plastic. Therefore, it is necessary to find ways to increase these properties and make these materials competitive, as also highlighted by Rodríguez-Felíx and colleagues [6].

The first method is to use plasticizing agents to increase elasticity. A plasticizer, such as polyols, fatty acids, polyethylene glycol and glycerol, acts on a polymer chain by increasing the free volume and improving chain mobility, also lowering the glass transition temperature [7]. The main difficulty of this method lies in identifying the right plasticizer concentration to use because, above a certain limit, there is the risk of obtaining the opposite effect, i.e., an increase in fragility [8]. A second method is cross-linking, to increase its tensile strength, thanks to the formation of inter- and intra-molecular covalent bonds between the polymer chains. This technique, particularly useful in the case of proteins considering their functional groups, can be carried out through the use of chemical agents (formaldehyde, glutaraldehyde, phenolic compounds), radiation treatment (UV or gamma rays), or through the use of enzymes (transglutaminase) [9,10,11]. In addition, it is possible to improve mechanical properties through the manufacture of composite films, multilayers, or polymeric blends, that allow the exploitation of the advantages of different polymers to create a product with better performance [12,13].

This work has taken into consideration the possibility of using a polymer blend to improve the properties of zein-based films intended for food packaging applications. In particular, chitosan was selected as a second natural polymer for the blend preparation.

Chitosan is a polysaccharide obtained through a process of deacetylation of chitin, the second most abundant polymer on earth after cellulose [14], which represents the main constituent of the exoskeleton of crustaceans and it is also present in the cell of fungi and insects [15]. Unlike zein, chitosan is a hydrophilic polymer particularly soluble in acidic solutions. In general, it is an excellent material thanks to its transparency, good film-forming capacity and antimicrobial character. In this regard, chitosan-based antimicrobial packaging is shown to have the potential to inhibit the activity of pathogenic microorganisms that contaminate foods and, therefore, to have the ability to prolong shelf-life, improving the quality of packaged foods [16]. In the work of Zhang et al., the production of the polymer blend of chitosan to extend the shelf-life of mushrooms was investigated, and the need to improve the mechanical and gas barrier properties was highlighted [17]. Nwabor et al. achieved a marked extension of the shelf-life of chicken sausages by working with a mixture of poly vinyl alcohol and chitosan containing silver nanoparticles as an additional antimicrobial agent [18]. Furthermore, chitosan-based films have been used by Eze et al., as materials capable of monitoring the spoilage of fish-based foods, in particular shrimps, through visible colorimetric changes correlated to pH variation [19]. The blend of zein and chitosan was also studied by Escamilla-García et al., which reports a detailed analysis of the structural changes induced by the mixture of the two polymers and which evidenced the effect of the polymer concentration variation on the properties of the resulting films [20].

In this context, the present study concerned the optimization of the polymeric blend film production, and an analysis of the characteristics obtained by the use of the blend in comparison to the properties of the films based on the pure natural polymers studied was conducted. The technique selected for the creation of the films was solvent casting. This technique has innumerable advantages, including cost-effectiveness, high productivity and the possibility of modulating the process temperature, which makes it suitable for processing natural polymers [21]. In particular, the materials obtained were characterized in terms of mechanical properties by means of tensile tests, surface properties by measuring the contact angle (CA) and oxygen barrier properties.

The novelty of this work lies in having improved the properties of polymeric films made with two biopolymers of great interest, zein and chitosan, using an economical and sustainable strategy, i.e., the production of a blend by solvent casting, using green and Generally Recognized As Safe (GRAS) solvents, and without the use of plasticizers usually employed to improve mechanical properties, where pure biopolymers are very scarce.

## 2. Materials and Methods

### 2.1. Materials

Purified zein, chitosan (medium molecular weight) and glycerol (>99.5%, liquid) were purchased by Sigma-Aldrich, Milan, Italy; ethanol and acetic acid glacial (extra pure) were provided by Carlo Erba Reagents, Milan, Italy.

### 2.2. Polymeric Blend Optimization for Film Production

The solvent casting technique was used to produce films of pure zein, pure chitosan and a zein/chitosan blend.

Zein solutions were prepared by testing different polymer concentrations: 20%, 25% and 35% *w*/*w* dissolved, under stirring at 40 °C, in a hydroalcoholic solution of ethanol 80% *v*/*v*, up to solutions of zein 2% by weight with respect to the ethanol solution at 90% *v*/*v*, following the suggestions reported in [17]. As a plasticizer, glycerol was added, at 4% *v/v* with respect to the volume of solvent, directly into the solutions considering the results obtained in previous work [22].

Zein films were produced by pouring 2.5 mL of the solution onto glass Petri dishes of 9 cm diameter and placed in an oven at 60 °C for 2 h before being detached.

Chitosan solutions were similarly prepared by dissolving different polymer concentrations equal to 1%, 1.5%, 2%, 3% and 4% *w/v* in a solution of acetic acid 10% *v/v* in deionized water, under stirring at 80 °C for 1 h. In this case, the glycerol was added both directly in the solution and by spreading only on the plates to favor the detachment of the film. A total of 5 mL of each solution were poured onto the plates and placed in an oven at 60 °C for one day and then in a desiccator before the detachment.

The polymeric blend solutions were prepared by combining the single polymer solutions, prepared separately, in different polymer weight ratios: 10:1, 5:1, 2:1, 1:1 and 1:2. The resulting solutions were left under stirring at 80 °C for another hour. A total of 5 mL of each blended solution were poured onto Petri plates, dried in an oven at 60 °C for one day and then left in the desiccator for another day before being detached. Glycerol was spread only on the plates to facilitate the film detachment. All tests were conducted at least 7 times.

**Figure 1:**
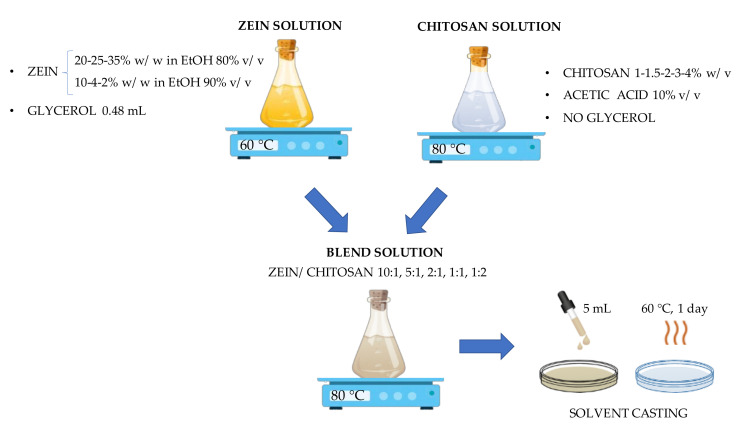
Summary of experimental test carried out for blend optimization.

Figure 1 shows a graphical schematization of the tests carried out for blend optimization.

### 2.3. Morphological Characterization

Ultra-high resolution field emission scanning electron microscope (UHR-FE-SEM, CrossBeam 1540 XB, Zeiss GmbH, Oberkochen, Germany) was used to analyze the morphology of the samples obtained, both the surface and the cross-section.

The film’s thickness was measured by a digital micrometer (3791G 0-150, Messzeuge, Spangenberg, Germany), recording 6 measurements for each sample.

### 2.4. Optical Properties

The optical properties of the films were evaluated in terms of transparency. The samples were cut into rectangles of approximately 10 mm × 45 mm so as to cover the entire internal surface of the cuvettes used for the test.

The samples were analyzed by UV-vis spectrophotometer (Lambda 25, Perkin Elmer, Wellesley, MA, USA) at 600 nm. Transparency was evaluated as the percent transmittance of light (%T_600_) measured at 600 nm following the suggestions reported in [23,24], and an empty cuvette was used as a reference. The tests were analyzed in triplicate, and the results were expressed as mean value ± standard deviation.

### 2.5. Mechanical Properties

Mechanical tests were conducted on all the samples produced using the Zwick Roell Z0.5 machine, (Zwick Roell S.r.l., Genoa, Italy) equipped with a 500 N load cell, in combination with Zwick Roell’s testXpert III software. Tensile strength (σ_M_), elongation at break (ε_B_) and Young’s modulus (E) were evaluated by tensile test according to ASTM638 standard method and UNI EN ISO 527 for polymeric films [25]. A tensile speed of 10 mm/min and an initial grip separation of 21 mm were used. At least six specimens of size 1 × 5 cm^2^ were tested per sample. The results were expressed as mean value ± standard deviation.

### 2.6. Oxygen Barrier Properties

The oxygen barrier properties of the films were determined in terms of permeability P [cm^2^/s], diffusivity D [cm^2^/s], and solubility S [-], using ultra-high vacuum laboratory equipment homemade assembled for P measurements on thin membranes. The measurements were carried out in accordance with the ASTM D1434-82 standard and following the procedures described in [26], applying a pressure difference ∆P equal to 10^5^ Pa. In detail, the permeability value was obtained from Equation (1) derived from Fick’s law:

(1) P =JLΔP

where ∆P is the difference between upstream and downstream pressure, J is the rate of transfer per unit area through a sample cross-section, and L is the membrane thickness. The diffusion was obtained by Equation (2), applying the method of the lag time, t_L_. Finally, S was obtained from Equation (3) under the hypothesis of Henry’s law using the following:

(2) D =L26tL

(3) S =PD

### 2.7. Thermal Analysis

The TG/DTA experiments were carried out in a nitrogen atmosphere using a STA 409 thermobalance (Netztch, Verona, Italy) with a sensitivity of ±0.1 mg, equipped with a Netztch 410 furnace temperature controller system. For each test, the samples were placed inside the furnace on a 6 mm diameter alumina crucible, and then the temperature was raised from room temperature to 800 °C at a nominal rate of 10 °C/min, with an intermediate isotherm step at 105 °C for 30 min to permit the complete drying of the sample.

### 2.8. Wettability

The wettability of the sample’s surface was investigated by the contact angle measurement using a video camera and goniometer-integrated equipment. The tests were carried out using a drop of distilled water at room temperature on the zein, chitosan and polymer blend films. The drop shape was recorded at instant zero and after 60 s to once a state of equilibrium was reached. At least 5 measurements per sample were conducted. The results were expressed as mean value ± standard deviation.

### 2.9. Statistical Analysis

Statistical analysis was performed by Statistica v8.0 software (StatSoft, Tulsa, OK, USA). Analysis of variance (ANOVA) and Tukey’s post hoc tests were used to assess the significance of differences among groups, with statistical significance considered at a probability value of *p* < 0.05.

## 3. Results and Discussion

### 3.1. Polymeric Blend Optimization

The solvent casting technique was used for the production of the polymeric films. Initially, the optimal solution composition and process conditions for obtaining transparent and defect-free films composed of pure zein and pure chitosan were investigated using a hydroalcoholic solution of ethanol at two different percentages (80% and 90% *v*/*v*) and acetic acid 10% *v/v*, respectively.

In the case of zein, the best solution and process conditions were the following: zein 20% *w*/*w* in 80% *v/v* ethanol, adding glycerol 4% *v/v* as a plasticizer and pouring 2.5 mL on Petri dishes and leaving in an oven at 60 °C for 2 h. Conversely, higher concentrations of zein, 25% and 35% *w*/*w*, led to the production of films that were too stiff and brittle, while lower concentrations led to the formation of films too thin to handle and analyze.

In the case of chitosan, different concentrations of the polymer were tested. The chitosan 4% *w/v* solution in acetic acid 10% *v/v* was too viscous to be processed, allowing us to identify the upper concentration limit for this polymer. The other less concentrated solutions were processed by pouring 5 mL and leaving them to dry in an oven at 60 °C for 24 h to obtain complete evaporation of the solvent. The 1% and 1.5% *w/v* chitosan films were too thin to be removed from supports smoothly, while the 2% and 3% *w/v* ones resulted in transparent thin films suitable for characterization.

Once the single films composed of pure polymers were optimized, the same solutions, prepared separately, were mixed in different polymer weight ratios (10:1, 5:1, 2:1, 1:1, 1:2) to produce the blended solution. In particular, working with zein solution at 20% *w*/*w* and chitosan at 2% *w*/*v*, mixed in a 1:1 weight ratio, the precipitation of the chitosan occurred with consequent separation of the two polymers once dried. Considering the upper concentration limit of chitosan (4% *w*/*v*), the concentration of zein was then lowered below 20% *w*/*w*. The best weight ratio of zein/chitosan polymers was found to be 1:2. This ratio was then kept constant, and the concentration of each polymer was increased to identify the optimal conditions for blend production, which were zein at 2% *w*/*w* in ethanol 90% *v/v* and chitosan at 4% *w/v* in acetic acid 10% *v*/*v* and pouring 5 mL of the resulting solution and spreading a little amount of glycerol directly on the Petri dishes rather than into the solution. It must be noticed that the low-concentrated zein solution decreased the viscosity of the pure chitosan solution, allowing the process of chitosan at higher concentrations. A summary of the experimental tests carried out and of the raw results obtained are summarized in Table 1. Furthermore, the best results were obtained by using ethanol at 90% *v*/*v*, probably due to the reduction of water volume in the resulting solutions.

### 3.2. Morphological Characterization

**Figure 2:**
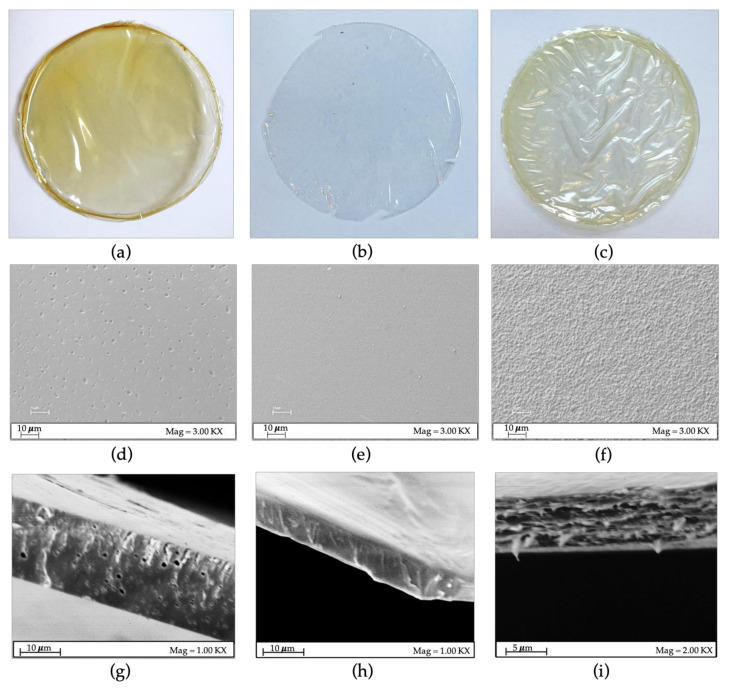
(**a**) Zein 20% w/w film sample, (**b**) chitosan 2% *w/v* film sample and (**c**) blend film sample. (**d**) Zein FE-SEM image, (**e**) chitosan FE-SEM image and (**f**) blend FE-SEM image. (**g**) Zein cross-section view, (**h**) chitosan cross-section view and (**i**) blend cross-section view.

The zein, chitosan and blended films produced are reported in Figure 2, with their relative morphology obtained by SEM analysis.

In particular, the zein films obtained (Figure 2a) are transparent and yellow in color due to the color of the commercial zein used, and the SEM images (Figure 2d) showed an almost periodic porosity also present in the thickness of the film, as evidenced by the cross-section view (Figure 2g), probably due to the fast solvent evaporation, as also evidenced by Bueno and colleagues [27]. The chitosan films present high transparency (Figure 2b) and a very smooth and uniform surface without defects (Figure 2e). In addition, looking at the cross-section, which highlights the continuity of the material (Figure 2h) while the polymeric blend presents an intermediate color and (Figure 2c), a rough surface with a uniform distribution of particles (Figure 2f) probably caused by the entanglement of the polymer chains of the two polymers, as also observed by Zhang et al. [17], and confirmed by the cross-section reported (Figure 2i). Furthermore, a shrinkage effect of the blended films is highlighted (Figure 2c), probably due to the polymer chains’ necking induced by the cooling phase after the detachment.

### 3.3. Optical Properties of the Films

The optical properties of the films were characterized by evaluating the transparency expressed as the percent transmittance of light (%T) measured by spectrophotometry at 600 nm, the intermediate wavelength in the visible range, and the one more easily comparable with the literature’s results. The tests were carried out using an empty cuvette as a reference sample. The prepared samples were carefully fixed inside the cuvettes so that they were perpendicular to the light beam.

**Figure 3:**
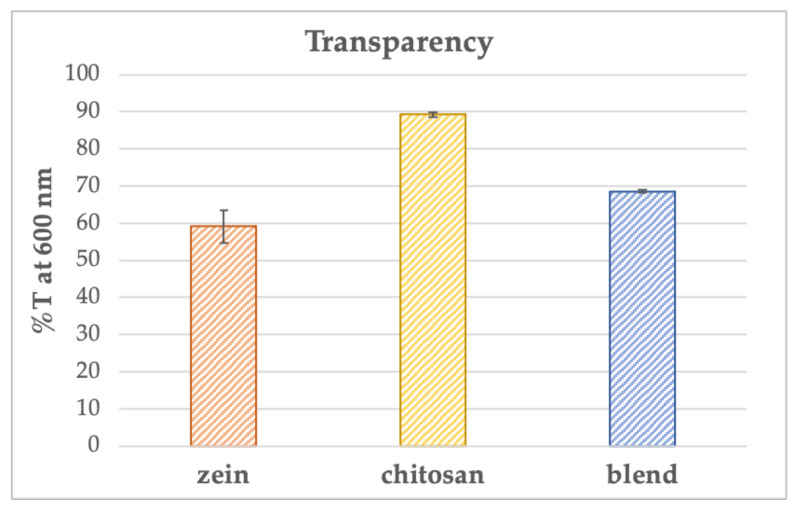
Transparency (%T_600_) of the films was evaluated via UV-vis analysis at 600 nm.

The test results are shown in Figure 3. In particular, the chitosan films, as can already be deduced from Figure 2, present the highest transparency values, %T equal to 89.2 ± 0.7%, above the limits set for packaging materials, i.e., 80%, reported in detail in [23], from which it can also be noted that the results are comparable with those obtained with biopolymers, such as polyhydroxyalkanoates (PHAs) and low-density polyethylene (LDPE). Instead, both zein films and blends presented %T values of 59.1 ± 4.4% and 68.6 ± 0.3%, respectively, below the limits of transparent materials, falling into the category of translucent materials. Therefore, zein films and blends, having lower transparency values, seem to offer better barrier properties against light transmission on foods.

A transparent packaging material certainly represents an advantage from a marketing point of view as the consumer is more inclined to purchase a product that is clearly visible inside the package, but, for foods subject to rapid deterioration, considering the negative influence of UV, it is better to prefer materials that can act as a barrier to light. The blend, in this particular context, seems to be able to satisfy both aspects, offering optical properties intermediate to those of zein and chitosan.

### 3.4. Mechanical Properties

For the measurement of the mechanical properties, six specimens for each sample of zein, chitosan and polymeric blend were analyzed by tensile test after measuring the films’ thickness. Tensile strength, elongation at break and Young’s modulus were evaluated to investigate the effect of the blend formulation with respect to the performance of pure zein and pure chitosan films. The results of the mechanical tests and the films’ thickness are reported in Table 2.

From the results obtained, it can be seen that the σ_M_ of all samples was successfully found to exceed the minimum limit required for packaging application, i.e., 3.5 MPa [28]. In detail, the zein films show high brittleness, as indicated by the low values of ε_B_ and high stiffness due to the high values of E. It is probable that these results, also considering the statistical analysis evidence and in addition to being attributed to the poor intrinsic mechanical strength of zein, are due to the quantities of glycerol used, which, if present in too high quantities, can act as an anti-plasticizer reducing the performance of the material. Chitosan films present the highest σ_M_ values, lower brittleness with ε_B_ five times higher than the one obtained in the zein sample, but higher stiffness than zein films. Finally, the polymeric blend allowed us to obtain intermediate σ_M_ values compared with pure polymer films. Furthermore, it was possible to note that, through the production of the polymeric blend, it was possible to improve the properties of pure zein and pure chitosan in terms of ε_B_, which is higher, and E, which in fact, was lower in the case of the blend.

**Figure 4:**
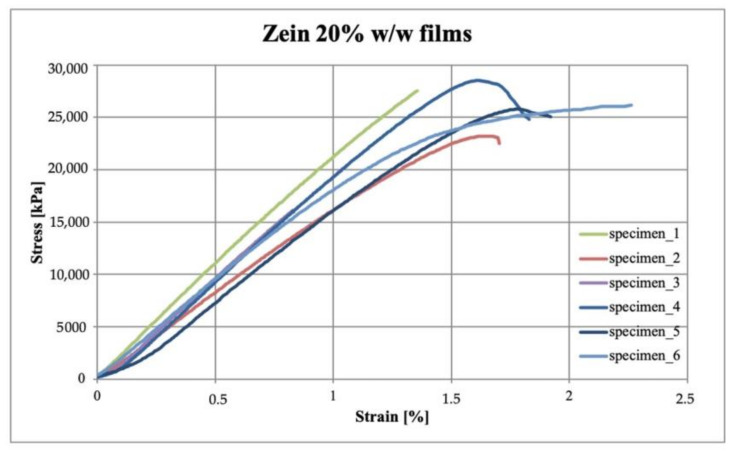
Stress–strain curves of zein 20% *w*/*w* films.

**Figure 5:**
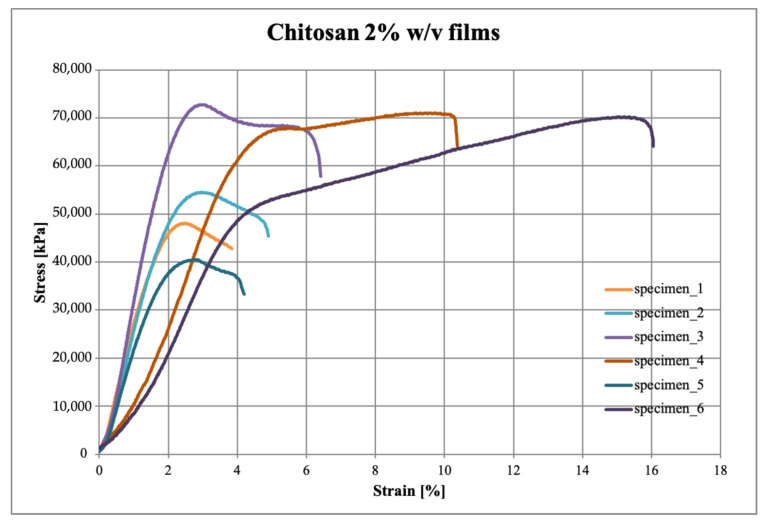
Stress–strain curves of chitosan 2% *w*/*v*.

**Figure 6:**
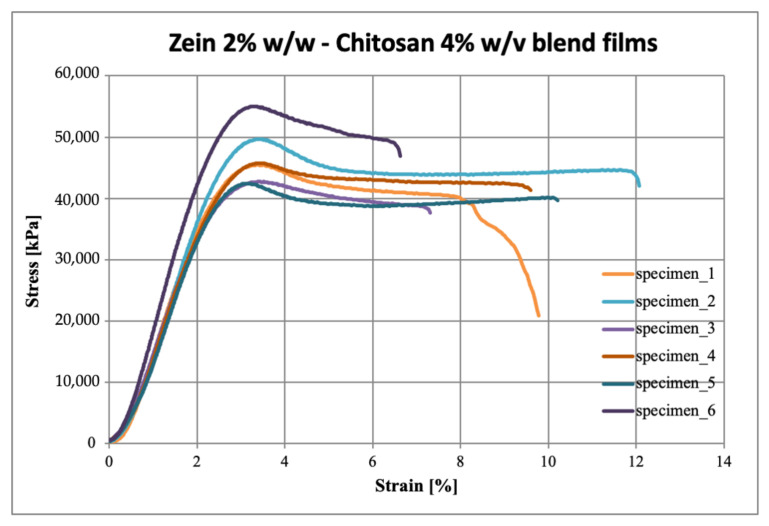
Stress–strain curves of zein 2% *w*/*w* and chitosan 4% *w/v* blend films.

The stress-strain curves obtained from the tensile test carried out on the six specimens per sample, are reported in Figure 4, Figure 5 and Figure 6 for zein, chitosan and blend films, respectively.

As can be observed, the zein presents a linear behavior typical of the brittle material, while chitosan and blend evidence a yield point and a plastic plateau. From Figure 5, it can be observed that the chitosan films show a more variable behavior, as also evident from the standard deviation values reported in Table 2, probably due to possible cracks introduced in the very thin samples during the specimen preparation phase. On the other hand, the blend films presented a more marked and uniform elastic behavior.

These results, comparable to those obtained by Yu et al. [29], showed that the production of a polymeric blend allowed to obtain less rigid, less fragile and more resistant films by processing small quantities of materials.

### 3.5. Oxygen Barrier Properties

The oxygen permeability tests were conducted as described in Section 2.5. In particular, considering the high pressures foreseen by the analysis, the area of each sample to be analyzed was evaluated, taking into consideration the thickness of each sample so as not to break the films. In the case of chitosan, the samples broke even when the area was minimized due to the very small thickness. The results obtained on the 20% *w*/*w* zein samples and the blend (2% *w*/*w* zein + 4% *w/v* chitosan) are shown in Table 3.

From the values reported in Table 3, it is possible to note how the P value decreases by at least two orders of magnitude compared with that obtained for the zein, i.e., the blend was found to be less permeable to oxygen. Additionally, in this case, the presence of glycerol in the zein may have contributed negatively to the barrier properties by acting on the mobility of the molecular chains.

These results highlighted how the polymer blend could actually improve some properties in which the single polymer does not excel. The diffusivity and solubility also decrease. In particular, the solubility of oxygen drastically decreases in the blend compared with pure zein, probably due to the lower chemical compatibility, i.e., a lower condensability of oxygen in the blend with a consequent reduction in permeability.

### 3.6. Thermal Analysis

**Figure 7:**
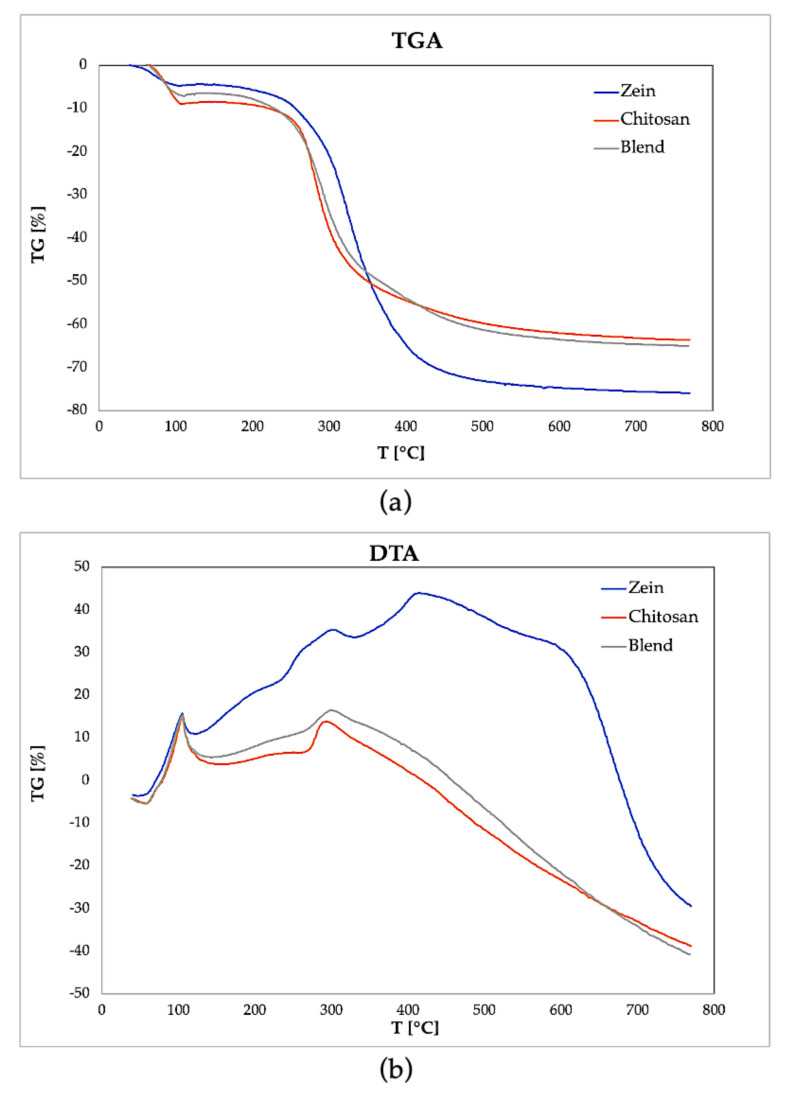
(**a**) Thermogravimetric analysis (TGA) and (**b**) differential thermal analysis (DTA).

The thermal stability of all the samples produced was analyzed via TG/DTA from 20 °C to 800 °C. The curves obtained are shown in Figure 7.

From the graphs, it can be observed that the initial weight loss of the samples due to moisture evaporation equals 4.5% for the zein film, 6.5% for the chitosan film and 8.5% for the blend. The thermal stability of the samples is maintained up to 230 °C, a temperature at which begin to appreciate a loss of weight and an onset of an exothermic peak in the DTA graphs (Figure 7b) due to the chemical degradation of the polymers. This exothermic peak is comparatively more prolonged in the zein, from 240 to 340 °C, while in the other two samples of chitosan and blend. It is appreciable starting from 270 °C. Furthermore, zein shows a second exothermic peak at 420 °C, probably due to the pyrolysis of carbon residues. The total weight loss is 75% for zein, comparable with the literature’s results [6], and 65% for chitosan and blend.

### 3.7. Wettability

**Figure 8:**
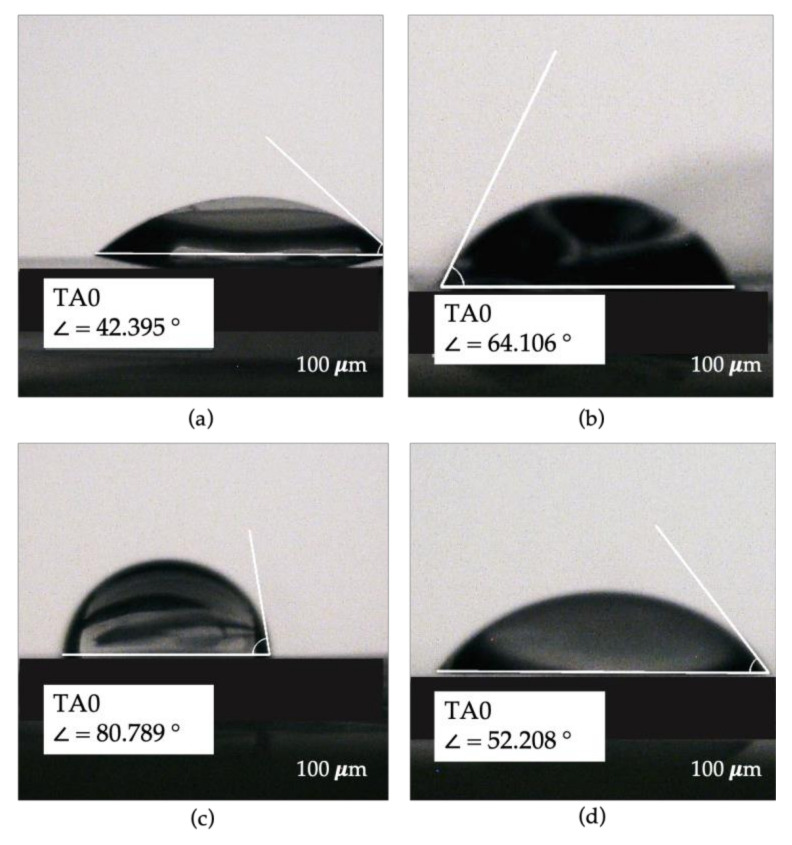
Contact angle (CA) obtained from (**a**) zein 20% *w*/*w* film, (**b**) chitosan 2% *w/v* film, (**c**) blend film at instant zero and (**d**) blend film after 60 s.

The sessile drop method was used to perform the CA measurement on all the samples produced. The test carried out highlighted a very interesting outcome, as shown in Figure 8, in which some photos of the CA are reported.

In detail, the films fabricated with zein, which is a hydrophobic protein constituted of non-polar amino acids [30,31], exhibit hydrophilic surface properties with a CA of less than 90° and equal to 42 ± 4.5° which remained stable over the test time, i.e., 60 s. Instead, the films realized with chitosan, a polysaccharide of hydrophilic nature, are confirmed to have hydrophilic surface properties presenting a CA of 67 ± 6.1°, but higher than the one measured on zein films. Finally, the produced blends show a CA of 77 ± 4.1° at instant zero, probably due to the rougher surface than the other specimens, with a variation in its shape up to 47 ± 10.6° after 60 s, highlighting a microcapillary phenomenon due to the higher surface roughness, as observed in Figure 2f. Therefore, by comparing results after stability was achieved, the production of the blend does not particularly modify the surface properties obtained from the pure polymers. In particular, the expected results of decreasing the hydrophobicity of the zein by mixing it with a hydrophilic polymer were not obtained. Hence, the surface properties of the material are not only associated with the raw polymer but also with the production technique and with the choice of the solvent used, as also discussed by Yu et al. and Yoshino et al. These authors argued that the use of acetone as a solvent for zein allows for maintaining its hydrophobic characteristics if high evaporation rates are used, while the use of acetic acid for zein allows for improving its mechanical performance in terms of less brittleness and stiffness [29,31].

Furthermore, the presence in the zein films of glycerol, a hydrophilic plasticizer, contributed to the modification of the surface properties of the samples obtained, as also reported in [32]. Thus, if hydrophobic characteristics are needed, it could be convenient to use hydrophobic plasticizers, such as oils and to change the solvent.

## 4. Conclusions

This work aimed to formulate a natural polymeric blend of zein and chitosan via solvent casting as a method to improve the physical performance of naturally sourced packaging materials using a simple and cost-effective process.

The blend composition used in this work resulted in the production of packaging materials with improved properties compared to pure polymer films. Positive results have been achieved in terms of optical properties. In fact, the blend showed better transparency values than pure zein and better light barrier properties than pure chitosan. In general, transparency has proven to be modular according to the desired purpose: in order to achieve greater transparency, it is advisable to increase the amount of chitosan, while to obtain a better UV barrier, zein becomes essential.

The blend production has improved the mechanical properties of the pure polymers, and the presence of chitosan in the blend has allowed operation without the need for plasticizers. The blend films showed better oxygen barrier properties with a permeability value of at least two orders of magnitude lower than that obtained for the pure zein.

Furthermore, the production of the blend did not affect the thermal stability of the samples and the wettability. Moreover, it was interesting to observe that, for the films produced with pure zein, a hydrophobic protein, contact angles of about 40° were obtained, highlighting the modifications induced by the solvent and by the presence of a hydrophilic plasticizer, such as glycerol, on the wettability.

In conclusion, this work has demonstrated how it is possible to obtain thin polymeric films starting from agro-industrial waste using small quantities of raw material, green solvents, and sustainable, cost-effective and scalable processes, such as solvent casting and the blend strategy, confirming the potential of these materials to become worthy substitutes for plastics.

## Figures and Tables

**Table 1:** Set of experimental tests was carried out for polymeric blend production and main raw results.

Zein [*w*/*w*]	Chitosan [*w*/*v*]	Results
20%	2%	Not blended
10%	2%	Not blended
4%	2%	Not film formation
2%	2%	Polymers separation
1%	2%	Too thin
1.5%	3%	Too thin
2%	4%	Good

**Table 2:** Mechanical properties of pure zein, pure chitosan films and blended films in terms of film thickness, tensile strength (_σM_), elongation at break (ε_B_) and Young’s modulus (E). The different letters show statistically significant differences at *p* < 0.05.

Sample	Thickness [μm]	σ_M_ [MPa]	ε_B_ [%]	E [GPa]
Zein	60 ± 8	25 ± 5 ^b^	1.6 ± 0.4 ^b^	1.9 ± 0.2 ^a^
Chitosan	15 ± 5	64 ± 11 ^a^	7.6 ± 4.7 ^a^	2.2 ± 1.0 ^a^
Blend	30 ± 8	47 ± 5 ^a^	9.2 ± 1.9 ^a^	1.8 ± 0.2 ^a^

**Table 3:** Oxygen barrier properties of pure zein films and blend films in terms of permeability (P), diffusivity (D) and solubility (S).

Sample	P [cm^2^/s]	D [cm^2^/s]	S [-]
Zein	(4.7 ± 0.4) × 10^−9^	(6.0 ± 0.7) × 10^−10^	7.6 ± 1.6
Blend	(1.3 ± 0.1) × 10^−11^	(1.4 ± 0.2) × 10^−10^	0.09 ± 0.02

## Data Availability

The data presented in this study are available on request from the corresponding author.
